# School-Based Intervention Programs for Preventing Obesity and Promoting Physical Activity and Fitness: A Systematic Review

**DOI:** 10.3390/ijerph17010347

**Published:** 2020-01-03

**Authors:** Hidayet Suha Yuksel, Fatma Neşe Şahin, Nebojsa Maksimovic, Patrik Drid, Antonino Bianco

**Affiliations:** 1 Faculty of Sport Sciences, Ankara University, 06830 Ankara, Turkey; 2 Faculty of Sport and Physical Education, University of Novi Sad, 21000 Novi Sad, Serbia; 3 Department of Psychology, Educational Science and Human Movement, University of Palermo, 90144 Palermo, Italy

**Keywords:** physical activity, obesity, physical fitness, sedentary behavior, intervention, school, health promotion, physical activity promotion

## Abstract

With the significant decrease in physical activity rates, the importance of intervention programs in the schools, where children spend a significant part of the day, has become indisputable. The purpose of this review is to systematically examine the possibility of school-based interventions on promoting physical activity and physical fitness as well as preventing obesity. A systematic approach adopting PRISMA statement was implemented in this study. Three different databases (2010–2019) were screened and primary and secondary school-based intervention programs measuring at least one variable of obesity, physical activity, or physical fitness were included. The risk of bias was assessed using the validated quality assessment tool for quantitative studies. Among 395 potentially related studies, 19 studies were found to meet the eligibility criteria. A general look at the studies examined reveals that among the outcomes, of which most (18/19) were examined, a significant improvement was provided in at least one of them. When the program details are examined, it can be said that the success rate of the physical activity-oriented programs is higher in all variables. School-based interventions can have important potential for obesity prevention and promotion of physical activity and fitness if they focus more on the content, quality, duration and priority of the physical activity.

## 1. Introduction

Physical activity (PA) is one of the most effective ways of preventing cardiovascular and mental illnesses and improving physical fitness (PF). Regular PA reduces the risk factors of many diseases such as high blood pressure, diabetes and obesity [1]. On the other hand, inactivity may cause obesity and a low level of cardiovascular fitness and increase the risk of high blood pressure and cholesterol with children. This situation also may lead to the development of chronic diseases such as high blood pressure and diabetes during adulthood [2]. In the systematic study of Janssen and Leblanc [3] where the relationship between PA, PF, and health of school-age children and adolescents were examined, it was found that PA contributed significantly to health, especially in high-risk youth, and that PA should be continued at least moderately to maintain health benefits and that vigorous activities provided more benefits. In another systematic study examining the interventions to increase moderate-to-vigorous (MVPA) PA in physical education classes, it was concluded that the intervention could increase MVPA levels throughout the course and provide significant benefits to public health [4].

To achieve health benefits, it is recommended to exercise with MVPA intensity for five days a week or more for at least 30 min, or with vigorous intensity for at least 20 min for 3 days [5]. The World Health Organization (WHO) [6] recommends MVPA for children and adolescents for at least 60 min per day. Despite the importance of regular PA, PA levels have started to decrease together with the new tools such as smartphones, computers, tablets, video games and social media, that have come into our lives in relation to technology [1]. PF of children has also declined with low levels of PA [7]. High levels of PF in children and adolescents are linked to physical and mental health [8]. At this point, the role of schools, where children and adolescents spend a significant portion of their days, in providing PA opportunities has reached an even more critical point. Despite this critical situation, the scope, quantity and quality of physical education courses, as well as the participation rates in extracurricular physical activities are not at the desired levels and differ significantly from school to school [9,10,11]. Schools are easy and provide accessible settings to promote engagement in PA because it is related to the objectives of the physical education curriculum. Children can learn the knowledge, skills and attitudes necessary for lifelong PA habits in schools. WHO [6] maintained that “all children and young people should be physically active with the support of schools or public institutions through games, sports, recreation, physical education and planned physical activities” and underlined the mission of schools regarding PA. School-based PA interventions include various categories such as physical education curriculum, classroom activity breaks, active commuting to school, modified playgrounds and comprehensive multi-component approaches. Along with learning in psychomotor, cognitive and affective domains, PA and PF levels will be promoted and obesity will be prevented over time. In this context, the priority is that children have meaningful learning experiences during their school years. Considering the seriousness of problems, which may occur related to inactivity in children, increasing school-based PA options of children and ensuring that children spend time with PA at schools should be considered a vital task.

Although PA programs in schools are one of the primary sources for promoting PA and FP, children are not active enough and their obesity/overweight levels have reached alarming levels [12,13,14,15]. A sedentary lifestyle and changing dietary habits have significantly increased obesity among children and adolescents [16]. Overweight and obesity are operationally defined as ‘‘abnormal or excessive fat accumulations that negatively affect health’’ [17]. WHO accepts childhood obesity as one of the most important global problems of the 21st century [18]. Overweight or obesity is a very complex phenomenon with many different causes. Although it is widely accepted that the increase in obesity stamps from the imbalance between energy intake and expenditure, it is known that there are many other genetic, environmental and behavioral reasons. The ecological model, as described by Davison et al., suggests that child risk factors for obesity include dietary intake, PA, and sedentary behavior [19]. In addition, environmental and socio-cultural factors such as family characteristics and parents’ lifestyle, school policies, and screen culture affect eating and activity behaviors [20]. Therefore, school-based intervention programs to prevent obesity should be designed and diversified in a comprehensive and multicomponent way. Physical inactivity is one of the most important factors in the emergence of obesity [21,22]. Traditional interventions for overweight or obesity include training in healthy nutrition and changing lifestyles by increasing PA. In this context, interventions based on increasing PA are often considered to be the most effective method, not only because they help in weight control but also because of health benefits such as the strengthening of bones and muscles, better sleep, improving mental health and reducing the risk of cardiovascular disease [3,23]. In a longitudinal study performed on more than 6000 children at the age of 7 years doing regular PA, it was concluded that performing PA was associated with the body fat percentage of children at the age of 11 years [24]. Again, researches conducted on children aged between 4 and 18 show that being engaged in regular PA has basic health benefits such as the increasing of bone density, good blood pressure and the improving of metabolic or cardiopulmonary health [3,25]. Apart from these, the benefits of PA can be stated as increased attention span, healthy functioning of cognitive processes, mental health and a general state of being healthy [26,27]. Regardless of the intensity, PA is associated with better health, motor skills and positive cognitive outcomes [28].

School-based PA is a necessary process that requires the structuring of all the time spent in the school, beginning with the planning of the child starting the day actively as s/he comes to school. In order to increase the participation of children in PA in schools and to prevent obesity effectively, it is necessary to increase the active time in physical education courses, to diversify the content of the course, to reevaluate the teacher qualifications and the number of students, to examine the effectiveness of programs for recess, in-school activity, extracurricular activities and school teams. Some systematic studies conducted in this respect have shown that school-based interventions can be effective in preventing obesity and promoting PA [29,30,31]. On the other hand, there are also studies in which variables such as obesity, PA and sedentary behavior are measured and no significant development can be observed [23,32,33]. In a significant part of the studies carried out in the literature, the differentiation of the success of situations in terms of the variables examined necessitates the examination of the studies conducted in this field from different aspects [34,35,36]. In this systematic review, it is aimed to contribute to the literature from different perspectives by including variables such as the content, type and duration of the physical activity which can directly affect the success of the school-based programs and are not sufficiently emphasized in other systematic review studies. Additionally, more studies are needed to evaluate the different contexts regarding the potential of school-based interventions. In this context, the study aims to systematically examine the possibility of school-based interventions for preventing obesity and promoting PA and PF.

## 2. Materials and Methods

**Figure 1:**
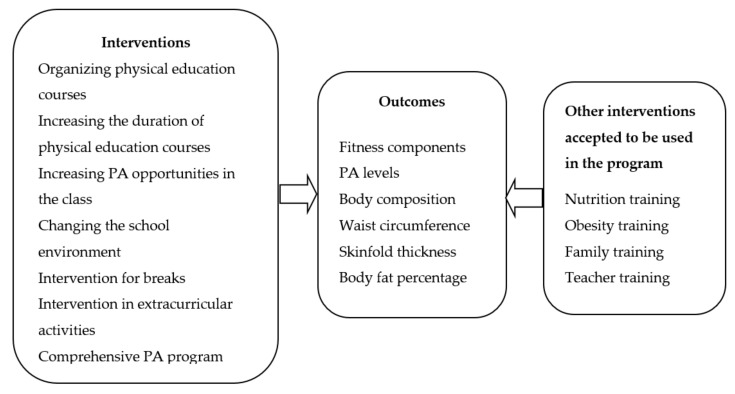
General framework of the systematic review. PA: Physical activity.

The framework used analytically by Kahn et al. [37] and modified by Demetriou and Honer [30] was organized and used particularly in this study. In this sense, the expected characteristics of the school-based interventions, the expected outcomes and the interventions that are accepted to be used outside of PA in such programs are presented in Figure 1. The systematic review does not include content that violates human rights, in compliance with the Helsinki Declaration; therefore, ethical approval was not needed.

### 2.1. Literature Search and Study Selection

In order to fully incorporate the relevant literature, a thorough electronic screening was conducted. The following databases were used to scan the data: (1) PubMed, (2) Web of Science, (3) Scopus. The scanning was focused on the following four key elements: population (children, adolescents), study design (trial), behavior (physical activity, walking, running, games, sports, etc.), and intervention (behavior change, higher BMI, level of physical activity, fitness). Focusing on these 4 elements, combinations of the terms school-based, physical activity, health, child, adolescent, intervention, overweight and obese were used in searching concepts (Supplementary File S2).

After the first screening based on databases, the title and abstracts were exported to an Excel file and records were screened by two researchers. At this stage, researchers excluded studies if they did not include physical activity and primary or secondary school students, and if it were not clearly indicated that the program was in a school setting. After the title and abstract review, the full texts of potentially related studies in the databases were recorded.

### 2.2. Eligibility Criteria

The main criteria used for inclusion in this study were the application of at least one of the programs presented in Figure 1 within the scope of school-based PA, and that the results of this intervention regarding at least one variable among the components of body composition, waist circumference, skinfold, PA level, and PF were measured. In addition to this, the studies consisted of primary or secondary school students as a sample and were published between the years 2010–2019, with English accepted as a criterion. Studies published in languages other than English were excluded. Besides, studies that did not take place within the scope of school-based PA and that did not involve intervention were excluded from the study even if the age group matched. The studies that did not include clear information regarding the measurement of outcomes about obesity and PA level or how PA was implemented were also excluded.

### 2.3. Data Extraction and Risk of Bias

The researchers initially summarized the abstracts of each article in the Excel file in order to serve the purpose of the research. At this stage, two different researchers examined the studies according to the criteria to be included in the study and articles that did not meet the criteria for inclusion in the study were extracted. The research design, population, intervention type, intervention details, data collection process, measured characteristics and findings of the studies remaining after this stage were filed. At the next stage, two independent researchers evaluated the quality of the full text of the studies through the “validated quality assessment tool for quantitative studies” developed in the Effective Public Health Practice Project (EPHPP) (Supplementary Table S1) [38].

The EPHPP quality assessment tool gives the six study components a strong, moderate or weak assessment (Table 1). Based on these ratings, a global quality rating is made. Powerful studies have no weak components. Moderate studies have only one weak component. Weak studies have two or more weak components.

## 3. Results

**Figure 2:**
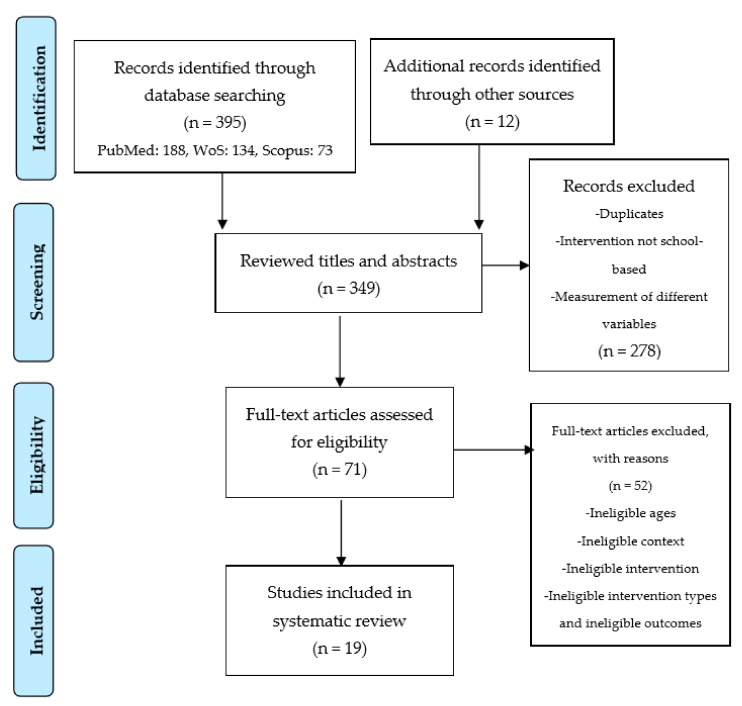
Flow diagram of studies through the review process.

In the first screening based on databases, 395 studies (PubMed: 188, WoS: 134, Scopus: 73) were obtained. After the exclusion of 58 repeated studies, 337 remaining titles and abstracts were read and evaluated by the researchers. In addition, systematic studies were examined, and the 12 studies found were included in the review process at this stage. After the title and summary review, the full texts of 71 studies in the databases were recorded. Nineteen studies were included in the final process after the studies that did not fit the criteria were excluded following the full-text review. The process was summarized and presented in the PRISMA flow diagram [39] (Figure 2).

Nineteen studies from 14 different countries (USA, Australia, England, France, Czech Republic, Iceland, Switzerland, Norway, Scotland, South Africa, China, India, Pakistan, Israel) met the eligibility criteria. 42.10% of these studies were identified as cluster randomized controlled trials, 21.05% as a non-randomized controlled trial, 15.78% quasi-experimental design, 10.52% randomized controlled trial and 10.52% as one-group, repeated measures design and mixed-effects model. An examination of the distribution of the schools studied revealed that 68.42% of the school-based intervention was realized in primary schools and 31.57% in secondary schools.

### 3.1. Description of Intervention and Study Quality

When the studies were evaluated according to the focus of the school-based program, the ratio of the physical activity-oriented studies (at least a direct intervention) was determined to be 73.68% and the ratio of the studies with education, guidance, counseling, and orientation support was determined to be 26.31%. The state of PA intervention being focused was evaluated according to whether it has a strategy or practice aimed at direct implementation of PA after the program details were examined. Among these studies, PA interventions have been in the form of increasing the duration of physical education or changing its content, performing extracurricular physical activities, doing activities during breaks or lunch breaks or giving activity breaks in the lessons other than physical education. In some researches, school-based interventions were in the form of examining the effects of a general project, while in some other researches it was in the form of directly intervening in a special group. In the control groups, nothing was changed. Two independent researchers helped to evaluate the quality of the studies. The results of the evaluation are given in Table 2. 

Besides, evaluation details are provided in the Supplementary Material. According to the evaluation results, 10 of the studies were evaluated as strong [40,47,49,50,52,54,55,56,57,58] and the remaining nine studies were evaluated as moderate. There were no studies evaluated as weak. In the general evaluation, the studies where two researchers made different decisions were discussed by them and the final decision was made. The efficiency and details of the studies are presented in Table 3 and Table 4.

### 3.2. Main Outcomes Regarding Obesity Parameters, Physical Activity and Physical Fitness

The studies revealed the main outcomes, including: BMI (kg/m^2^), waist circumference, skinfold and body fat percentage related to obesity parameters [40,41,42,43,45,46,47,49,50,51,52,53,56], the level of PA [45,48,49,50,51,52,54,55,57,58] and various variables related to PF components [42,49,52,53,54,56] during school years. Measurements of MVPA recommended in these studies were interpreted by measuring with the survey method in some studies [40,50,57] and by way of pedometer or accelerometer in some others [44,45,47,48,49,51,52,54,55,58]. In the studies where BMI (kg/m^2^) measurements were reported, it was found that body mass index was measured by taking height and weight measurements with standard tools. Three studies were also interpreted by measuring waist circumference [42,46,56]. Skinfold measurements were also performed in three studies [49,50,57]. In these studies, results regarding variables such as aerobic endurance, flexibility, muscular strength, muscular endurance, power, and speed which belong to fitness components were stated [42,45,49,52,53,54,56]. See Table 4 for details.

#### 3.2.1. Obesity

Although the values considered in the studies conducted within the scope of school-based intervention are all related to obesity, in this section, it will be focused on the studies where variables such as BMI, waist circumference and skinfold are measured, which may give a clearer idea. In this sense, body composition was evaluated in 15 studies [40,41,42,43,45,46,47,49,50,51,52,53,56,57,58]. In addition, in eight studies, body fat percentage was calculated by skinfold thickness, waist circumference thickness, or digital measurements [41,42,46,49,50,53,56,57]. While in eight of 15 studies in total (53.33%) there was a significant difference in favor of the intervention group in terms of BMI variable [40,41,43,45,47,49,50,58], there was no significant difference in six of them (40%) [42,51,52,53,56,57]. In the study conducted by Grydeland, Bjelland, Anderssen, Klepp, Bergh, Andersen, Ommundsen and Lien [46], while a significant difference was found in girls, no significant difference was found in boys (*p* > 0.05).

Significant improvement was observed in four (50%) of the eight studies (skinfold, waist circumference or digital measurement) in which body fat percentage was measured [41,42,49,50]; no significant difference was found in three studies (37.5%) [46,56,57]. However, while no significant difference was found in waist circumference and skinfold values in the study conducted by Magnusson, Hrafnkelsson, Sigurgeirsson, Johannsson and Sveinsson [53] (*p* > 0.05), the difference between the increases in fat percentage was found to be significant in favor of the experimental group (*p* < 0.05).

When the BMI variable was examined in studies more focused on PA, the success rate was 72.72% (8/11) [40,42,43,45,46,47,49,50,52,56,57]; and the success rate was found to be 50% (2/4) in studies using PA only as support [41,51,53,58]. These results indicate that physical activity-oriented interventions are more likely to be successful in the BMI variable.

Obesity is also a variable directly related to the duration of intervention. In this context, when the studies lasting less than 6 months and the studies lasting longer than 6 months are compared according to the duration of the intervention [40,41,42,46,49,52,53,56], the success rate of the BMI variable was 62.5% (5/8); in studies less than 6 months [43,45,47,50,51,57] this rate was 66.66% (4/6). This result shows that shorter interventions can achieve similar success in the BMI variable.

There are 11 studies where children who are obese or overweight are in the study group [41,42,43,46,47,48,49,50,53,56,58]. While significant progress was made in the intervention group in six of these studies [41,43,47,49,50,58], no significant difference was found in five studies [42,46,48,53,56].

In brief, it is understood that in almost half of the studies, there was an improvement in BMI (53.33%), waist circumference or skinfold (50%) values. It was determined that, in line with the type of intervention, the BMI variable improved significantly in 72.77% (8/11) of the physical activity-oriented studies and that a success rate of 50% (2/4) could be achieved in interventions focused on training, guidance, orientation or providing support.

#### 3.2.2. Physical Activity

Measurements regarding PA level were made in 13 studies [42,44,45,47,48,49,50,51,52,54,55,57,58]. In three of these studies, the evaluation was based on surveys, [42,50,57], a pedometer was used in four studies [45,51,54,58] and an accelerometer was used in five studies [44,47,48,49,52]. In the study conducted by Sigmund, El Ansari and Sigmundova [55], both accelerometer and pedometer were used. Since a substantial part of these studies was measured by objective methods, it was easier to evaluate them. In a substantial number of the studies conducted (61.53%), it was concluded that there was a significant increase in the PA levels of children compared to the control group [42,44,45,49,50,54,55,58].

Apart from that, while PA levels increased significantly in seven of the studies with the physical activity-oriented intervention [42,44,45,49,50,54,55], no significant development was observed in three of them [47,52,57]. Whereas PA levels increased significantly in one of the studies predominantly based on education and orientation [58], no significant increase was observed in the other two studies [48,51]. In addition, while PA levels increased significantly in two of the studies conducted through surveys [42,50]; the number of studies showing a significant increase in the studies measured by pedometer or accelerometer was determined to be four [49,54,55,58]. When evaluated in terms of duration, while a significant increase was observed in three of the studies where the intervention period lasted 6 months or less [45,50,54], no significant increase was observed in one study [51]. In the studies, which lasted over 6 months, while a significant increase was found in PA levels in five studies [42,44,49,55,58], development was not found to be significant in four studies [47,48,52,57].

In brief, although the type and extent of the interventions were different, PA levels of children increased in a significant proportion of the studies. While 70% (7/10) of the physical activity-oriented studies showed a significant increase, that 33.33% (1/3) success rate was achieved in studies focused on training and orientation. This result demonstrates the importance of applying PA strategies at the focus of the intervention program to increase the levels of PA.

#### 3.2.3. Physical Fitness

In seven studies examined, components related to fitness were analyzed [42,45,49,52,53,54,56]. As the first variable, the property of aerobic endurance was evaluated in terms of shuttle run, 1-mile run test and ergometer bike. It was concluded that in four of the studies examining the aerobic endurance, this variable increased significantly compared to the control group [45,49,53,56]. No significant development was observed in two studies [52,54]. Considering the type of intervention, significant improvement was observed in four intervention physical activity-oriented studies [42,45,49,56] and no significant improvement was observed in terms of aerobic endurance in two studies [52,54]. In the study, which was focused on education and orientation, significant developmental findings were found [53]. When evaluated in terms of intervention duration, the rate of studies that provided significant improvement in aerobic endurance in the intervention performed at 6 months or less was determined to be 66.66% (2/3) [45,56]. No significant difference was found in the study conducted by Shore, Sachs, DuCette and Libonati [54]. In studies with intervention over 6 months, the rate of studies, in which aerobic capacity increased significantly, was determined to be 66.66% (2/3) [49,53]. In the study conducted by Madsen, Linchey, Gerstein, Ross, Myers, Brown and Crawford [52], the change in the aerobic capacities of children was not found to be significant (*p* > 0.05).

Apart from aerobic endurance, four studies, most of which belong to health-related PF components, were examined in variables such as flexibility, speed, power, muscular strength and muscular endurance [42,45,54,56]. Among these, in the study conducted by Bhave, Pandit, Yeravdekar, Madkaikar, Chinchwade, Shaikh, Shaikh, Naik, Marley-Zagar and Fall [42], there was a significant improvement in favor of the intervention group in running, long jump, sit-up and push- up tests. In the study conducted by Eather, Morgan and Lubans [45], different variables related to PF were evaluated and significant improvement was observed in flexibility and muscular fitness properties in favor of the intervention group. In another study of Shore, Sachs, DuCette and Libonati [54], measurements were made regarding muscular strength, endurance and flexibility, but no significant difference was found compared to the control group. In the study where anaerobic capacity was measured in addition to health-related components, Thivel, Isacco, Lazaar, Aucouturier, Ratel, Dore, Meyer and Duche [56] found a significant difference in anaerobic fitness capacity in favor of the intervention group.

In brief, it is seen that in interventions based in school, measurements regarding all the health-related PF components (body mass index was examined in previous sections) were performed. Significant improvements were found in most of the studies predominantly focusing on aerobic capacity (66.66%). Improvements were observed in two of the three studies examining the different components of health-related PF. These improvements related to health-related PF parameters are valuable in terms of highlighting the importance of PA in improving children’s health.

## 4. Discussion

The purpose of this systematic review is to examine the potential of school-based interventions for obesity parameters (BMI, waist circumference, skinfold), PA level, and PF. Overall, 18 of the 19 studies examined were able to achieve significant improvements in at least one variable. It can be said that the content and details of the school-based intervention program are the most important factors in determining the efficiency in the studies examined. When the studies were classified according to the focus of PA in the program details, in 14 studies a strategy for direct PA and focus within the program was found [40,42,43,44,45,46,47,49,50,52,54,55,56,57]. Additionally, in five studies, it was understood that the education, guidance, orientation, and support in material of the staff and teachers in subjects such as health, nutrition and healthy life were in focus [41,48,51,53,58]. When the variables examined are considered, it can be said that the success rates in the physical activity-oriented studies are higher in all variables compared to those that are multicomponent but not centered on PA. This reveals the necessity of centralizing strategies aimed at PA to prevent obesity, promote PA and PF. Of course, the priority of programs for lifelong PA and health should be to prepare appropriate learning environments. Health-based physical education curriculum models aiming at a physically active life lead to the preparation of appropriate learning environments [59]. However, the relationship between the curriculum and pedagogy needs to be well understood in order to adopt more available approaches and not to neglect the enjoyable aspects of school-based PA [60]. From a holistic perspective, school-based PA programs can be more successful in promoting PA and PF. Castelli et al. [61] listed some of the properties that schools should have in order to educate physically active children: (a) a holistic approach and (b) effective and diversified pedagogy. Measuring of multicomponent interventions involving environmental and educational strategies is a complex and challenging process [62]. In this sense, although evaluations of different variables were made in school-based PA studies, only variables related to physiological dimensions were considered in this systematic research in line with the purpose.

Health promotion was the primary emphasis in school-based intervention programs. When health was evaluated from the physical aspect, the importance of school-based PA programs in improving health was revealed in this study. Similarly, in the systematic review made by Naylor et al. [63], most of the school-based intervention studies (11/15) were found to be positively associated with at least one health outcome. Of course, any program structured so as not to compromise health in relation to PA will contribute positively to health, but programs that are more comprehensive, centered on PA and games can be more helpful in this sense. In support of this situation, in the systematic study conducted by De Bourdeaudhuij et al. [64], it was suggested that the school environment should be rendered appropriate for PA, and PA durations should be increased in order to promote health. It would be more appropriate to integrate school-based programs into curriculum models in health promotion [59]. Curriculum models will ensure the persistence of effective and cognitive learning, which is crucial for the development of healthy living habits [65,66].

In eight of the 15 studies that evaluated obesity-related parameters, significant improvements were observed in variables such as BMI, skinfold, waist circumference and body fat; in seven studies, no significant development was observed regarding the whole group. In this sense, failure to achieve full success in almost half of the studies (46.66%) reveals that both the applied program and environmental conditions should be re-evaluated. Regarding this issue, in some systematic studies examining the effect of school-based PA on obesity parameters, it was concluded that it has no positive effect [23,67]. Although PA interventions are of great value in terms of short-term benefits in the fight against obesity or overweight, comprehensive programs are needed to have healthy living habits in the long term. At this point, it was understood that in most of the studies conducted to prevent obesity, multicomponent programs focusing on more than one dimension were applied [64,68]. These multicomponent programs should focus on content, teacher training and curriculum design to help children learn knowledge, skills and attitudes. In the study conducted by Brown and Summerbell [69], it was stated that the combination of programs based on nutrition and PA might be more effective in the struggle with obesity in the long term. For a more effective and permanent solution against the struggle with obesity, such comprehensive programs should include at least 60 min of MDVA, and interventions should be aimed at healthy diet and healthy living habits. Regarding the duration of the intervention, there was no difference in efficiency between the studies that lasted less than 6 months and more. In a meta-analysis by Harris et al., the shorter or longer duration of the intervention did not change the outcome of obesity [23].

Significant reductions in PA levels increase the importance of schools in providing a diversity of PA. In this sense, the use of different types of physical activities in school can increase children’s interest and encourage them to be more active [70,71]. In our study, improvement has been achieved in eight (61.53%) of the 13 studies aimed at increasing the level of PA. Similarly, in the systematic compilation conducted by Demetriou and Honer [30], it was concluded that in PA interventions in the school environment, 56.8% success was achieved in terms of increasing the level of PA. In the studies, in which PA intervention was predominantly in the center, development success on PA level reached higher rates (70–33.33%). An increase in the level of PA is important in every aspect, but when considered in relation to health, it is also important to indicate the intensity of PA. PA for at least 60 min per day with MDVA is suggested in order to improve health [3,6]. As interventions are very heterogeneous in terms of type, intensity and extent, there are difficulties in evaluating them at the point of MVPA. However, school-based PA programs have the potential to provide MVPA with high-intensity activity types [4]. In some of the studies evaluating the intensity of PA, interventions were found to be associated with MVPA [44,48]. Fairclough and Stratton stated that students engaged in MVPA for 27% to 47% of physical education class time [72]. To increase the intensity and duration of PA, it should be ensured that children are active when coming to school in the morning, during the break time, lunch break, in other lessons or at the extracurricular activities other than the physical education class. Physical education can be an effective influence on promoting PA [73]. Physical education courses should be the most complementary part of school-based PA, but this course only is not sufficient and more comprehensive programs are needed for lifelong activity [73]. The use of school-based interventions to increase the PA level of today’s children is very valuable. Some studies clearly demonstrate this potential [74,75].

The last variable considered in the study was the concept of PF. In seven of the studies conducted on this subject, components related to PF were evaluated and positive developments were observed in many of these studies. The primary objective of school-based PA programs is to reach and maintain health-related PF. Therefore, in the studies reviewed, the focus was rather on health-related fitness components. These are factors including components such as health-related PF, BMI, cardiorespiratory endurance (aerobic endurance), muscular strength, muscular endurance and flexibility [8]. While the feature that is mainly desired to be improved in the studies examined is aerobic endurance, it was found that there were also evaluations regarding the other components. In some studies, conducted in this sense, there are findings concerning the potential of school-based programs in improving PF [29,76]. There are also studies demonstrating the potential of the developed curricula for fitness education [59,77]. The results of our study show that even if the programs are multicomponent, the way of PA intervention is the most decisive factor at this point. In this sense, PA and games are essential for promoting PF in such programs [78].

The problems in evaluating the efficiency of such studies include: (1) the presence of very heterogeneous groups in general, and (2) the implementation of many interventions without adequate supervision, focusing only on guidance in schools, not long-term, and some of the measurement methods used are not objective. In order to prevent these problems, programs should be continued from preschool to the end of high school so that children have lifelong healthy living habits and they also should be based on a theoretical framework. The results obtained indicate that school-based intervention programs can have important potential for preventing obesity and promoting PA and PF if these problems are overcome and they focus more on PA.

The limitations of this study can be classified as follows:

(1) In this research, publications other than articles (theses, papers, books, etc.) and studies published in languages other than English were not included.
(2) Although many variables are measured in school-based PA programs, it was focused only on measurements related to obesity, PA, and PF in this study.
(3) Since the contents and the type of implementation of PA programs are generally multi-component, it is difficult to understand what the most effective way is in such studies.
(4) Evaluation of the focus of school-based intervention programs was determined according to the details given in the articles.
(5) Besides, some variables were measured by questionnaire and some studies did not provide enough details.
(6) In this study, variables such as curriculum, teaching models and teacher competencies related to the learning process in PA, and PF developmental processes were excluded.

### Practical Implications

The main purpose of the studies related to school-based PA programs and should be used to develop the motor skills necessary for children to be active throughout their lives, and also to educate children who exhibit positive social behaviors while improving their PA and fitness levels. In this sense, physical education teachers such as school sports leaders should consider different learning domains and include administration, family and other courses in the programs. Health professionals or experts should carry out PA interventions with sports scientists. In most of the studies examined, it was seen that the intensity of exercise was not planned before the interventions. In order to increase health-related contributions, interveners need to pay attention to this issue. Nowadays, school time is more precious than ever. Sports scientists should have more contact with this field and participate more actively in designing school-based intervention programs.

## 5. Conclusions

School-based interventions can have important potential in the health promotion of children in terms of obesity, PA level and PF. However, the quality, duration, and priority of PA intervention in comprehensive school-based programs and teacher capacity are some of the most important factors for preventing obesity and promoting PA and PF. If more impact is desired in school-based intervention programs, the focus of the program should be PA and, as far as possible, physical activities should be implemented directly. To prevent obesity and to promote PA and PF, the characteristics of more effective programs should be examined in detail and appropriate intervention programs should be designed. Such programs should be multicomponent and longitudinal to foster children’s lifelong PA habits. This habit requires knowledge, skills and attitudes. Our study focuses on the aspect of skill (physical). Furthermore, future studies should also focus on knowledge and attitudes in order to demonstrate holistic understanding. At the same time, for children to adopt a habit of PA, issues such as motivation, physical competence and interaction with the environment, which are at the basis of the cycle according to physical literacy, are also important. In this sense, the role of school-based interventions in improving children’s physical literacy should also be examined.

## Figures and Tables

**Table 1:** Quality assessment for studies.

Quality Assessment Tool for Quantitative Studies	
(A) Selection bias	Do the individuals selected for the study represent the target population? Number of individuals participating in the study.
(B) Study design	Is the study defined as randomized? If yes, is the randomization method specified? If yes, is the method appropriate?
(C) Confounders	Are there significant differences between pre-intervention groups? If yes, is the proportion of situations that cause confusion about the design or analysis indicated?
(D) Blinding	Are the assessors aware of the intervention or the exposure of the participants? Are the study participants aware of the research questions?
(E) Data collection methods	Is the validity of the data collection tools shown? Has the reliability of data collection tools been demonstrated?
(F) Withdrawals and dropouts	Were those who quit or were unable to complete reported? Is the proportion of those completing the study indicated?

**Table 2:** Summary of school-based intervention studies.

Study	Design	Setting and Participants	Aim	Intervention Duration	Quality
**Almas et al. [40]**	Parallel cluster intervention trial	4 public schools in Karachi, Pakistan. 277 girls (IG: 131, CG: 146).	To examine the feasibility of the school-based program and its effect on blood pressure and body mass index among pre-adolescent girls.	Over 20 months	Strong
**Aperman-Itzhak et al. [41]**	A non-randomized controlled trial	2 religious and 2 secular schools in Israel. 396 fifth- and sixth-grade students (IG: 200, CG: 196).	To evaluate the effectiveness of a healthy lifestyle intervention on health knowledge, behavior, and anthropometric measurements.	2011–2012 school year	Moderate
**Bhave et al. [42]**	A non-randomized controlled trial	Two schools in the cities of Pune and Nasik, India. 491 children (IG: 304, CG: 187).	To examine the 5-year impact of the program on preventing fat and improving PF and lifestyle.	5 years	Moderate
**Brown et al. [43]**	A one-group, repeated measures design	3290 children aged 4–12 years from southwest Scotland.	To examine body mass index standard deviation score changes following a Child Healthy Weight pilot intervention.	10 weeks	Moderate
**Carlson et al. [44]**	Mixed-effects model	Six elementary-school districts in California (n = 1322).	To examine the relationship between PA breaks and PA and class behaviors.	2013–2014 school year	Moderate
**Eather et al. [45]**	Randomized controlled trial	Four primary schools in the Hunter Region, NSW, Australia. 213 children (mean age = 10.72 ± 0.6; %52 female) (IG: 118, CG: 95).	To evaluate the impact of a school-based PA intervention (Fit-4-Fun) on health-related fitness.	8 weeks	Moderate
**Grydeland et al. [46]**	Cluster randomized, controlled study	12 schools in Norway. 1324 students (11-year-old) (IG: 465, CG: 859).	To examine the effects of a multicomponent school-based intervention on anthropometric outcomes.	20 months	Moderate
**Hollis et al. [47]**	Cluster randomized controlled trial	10 secondary schools in New South Wales, Australia. Baseline- IG: 645, CG: 505. Mid-point (12 months)- IG: 592, CG: 459. Follow-up (24 months)- IG: 560, CG: 425.	To report the secondary outcomes of the study; to determine whether the intervention impacted on adiposity outcomes (weight, body mass index (BMI), BMI z-score.	12 months	Strong
**Kipping et al. [48]**	Cluster randomized controlled trial	60 primary schools in the southwest of England. 2221 Primary school children (IG: 1064, CG: 1157).	To investigate the effectiveness of a school-based intervention to increase PA, reduce sedentary behaviour, and increase fruit and vegetable consumption in children.	5 years	Moderate
**Kriemler et al. [49]**	A cluster randomized controlled trial.	28 classes from 15 elementary schools in Switzerland. 502 children (IG: 297, CG: 205).	To assess the effectiveness of a school-based PA program during one school year on physical and psychological health in children.	9 months	Strong
**Li et al. [50]**	Non-randomized controlled trial	Four public schools in Changping District, Beijing of China. 921 children aged 7 to 15 years (IG: 388, CG: 533).	To assess the effectiveness of a school-based PA intervention during 12 weeks on obesity and related health outcomes in school children.	12 weeks	Strong
**Lynch et al. [51]**	A cluster randomized controlled trial	8 classrooms of second- and third-grade children in Rochester, Minnesota. 51 children (IG: 29, CG: 22).	To evaluate the impact of the Let’s Go! 5-2-1-0 program in an elementary school.	8 weeks	Moderate
**Madsen et al. [52]**	A cluster randomized controlled study	Six schools (IG: 4 schools, CG: 2 schools) in northern California. 879 students (IG: 583, CG: 296).	To examine the impact of Energy Balance for Kids with Play (EB4K with Play), on students’ PA, dietary habits and knowledge, and weight status over 2 years.	Over 2 years	Strong
**Magnusson et al. [53]**	A cluster randomized controlled trial.	Three schools in the city of Reykjavik, Iceland. 321 students (IG: 151, CG: 170).	To assess the effects of a 2-year intervention program on body composition and objectively measured cardiorespiratory fitness.	2 years	Moderate
**Shore et al. [54]**	A quasi-experimental design	One public middle school in a suburb near Philadelphia 92 students (IG: 46, CG: 46).	To determine the effects of a school-based pedometer intervention (SBPI) on daily accrued steps, academic performance, attendance, tardiness, and fitness performance in middle school students.	6 weeks	Strong
**Sigmund et al. [55]**	Non-randomized controlled trial	Four schools (2 control, 2 intervention) in the Czech Republic. 175 students (IG: 88, CG: 87).	To investigate the effect of increased PA on increasing daily PA and decreasing obesity in 6–9-year children.	2006–2008	Strong
**Thivel et al. [56]**	Randomized intervention study	19 primary schools in France. 457 children aged 6 to 10 years (IG: 229, CG: 228).	To assess the effect of a PA program on body composition and PF.	6 months	Strong
**Tian et al. [57]**	A pre-test and post-test control-group design	Two primary schools in Potchefstroom, South Africa. 110 Grade 7 learners aged 12–13 years (IG: 40, CG: 70).	To evaluate the effects of a once-a-week enhanced quality PE program on the PA levels.	Over 12 weeks	Strong
**Vander Ploeg et al. [58]**	Quasi-experimental, pre-post-trial with a parallel, non-equivalent control group	20 schools in Edmonton, Alberta, Canada. 1157 students in the year 2009 (IG: 198, CG:454), the year 2011 (IG: 196, CG: 309).	To examine the 2-year change in PA during and after school among students participating in a comprehensive school health (CSH) intervention.	2008–2011	Strong

IG: Intervention group; CG: Control group; PF: Physical fitness.

**Table 3:** Summary results of studies.

Study	BMI (kg/m^2^)	Body Composition	Physical Activity Levels	Physical Fitness
**Almas et al. [40]**	+	NA	NA	NA
**Aperman-Itzhak et al. [41]**	+	+	NA	NA
**Bhave et al. [42]**	−	+	NA	+
**Brown et al. [43]**	+	NA	NA	NA
**Carlson et al. [44]**	NA	NA	+	NA
**Eather et al. [45]**	+	+	+	+
**Grydeland et al. [46]**	+ (Only girls)	−	NA	NA
**Hollis et al. [47]**	+	NA	−	NA
**Kipping et al. [48]**	NA	NA	−	NA
**Kriemler et al. [49]**	+	+	−	+
**Li et al. [50]**	+	+	+	NA
**Lynch et al. [51]**	−	NA	−	NA
**Madsen et al. [52]**	+	NA	−	NA
**Magnusson et al. [53]**	−	−	NA	+
**Shore et al. [54]**	NA	NA	+	−
**Sigmund et al. [55]**	NA	NA	+	NA
**Thivel et al. [56]**	−	−	NA	+
**Tian et al. [57]**	−	−	−	NA
**Vander Ploeg et al. [58]**	NA	NA	+	NA

The “+” symbol indicates significant difference, the “−” symbol indicates no significant difference, and the abbreviation “NA” indicates that the relevant variable has not been examined in the study.

**Table 4:** Details of the studies.

Study	Types and Intensities of School-Based Programs	Outcomes Measured	Results				Multiple Components
**Almas et al. [40]**	**Type: PA program** Warm-up (The initial 5 min period) 20 min of aerobics The last 5 min comprised cool-down exercises. **Intensity:** 20 min MVPA	**PA:** Questionnaire (Last 7 days) **BP:** Omron M5 BP monitors **Height:** Community-setting aluminum scale **Weight:** Tanita Solar Powered Digital Scale 1631 **BMI = kg/m^2^**	**BMIz** Intervention	**BMIz** Control			
			Baseline Mean (SD): −1.35 (1.39) Follow-up Mean (SD): −1.02 (1.41)	Baseline Mean (SD): −1.92 (1.82) Follow-up Mean (SD): −1.04 (1.23)			
			The difference between systolic blood pressure (SBP), diastolic blood pressure (DBP) and BMI z scores (BMIz) of the experimental group and control group was found to be significant (1.9 mm Hg, 0.7 mm hg and 0.55 kg/m^2^).				
**Aperman-Itzhak et al. [41]**	**Type: A healthy lifestyle intervention** Eating a healthy breakfast, drinking water, PA, and reading food labels. Safe and attractive playgrounds Active play during break times **Intensity:** NA	**Healthy Lifestyle:** A self-administered non-quantitative FFQ **BMI (kg/m^2^):** Height, weight, and fat percentiles were measured using a Tanita BC 418MA Segmental Body Composition Analyzer	Overweight and obesity decreased significantly within the intervention group (from 25% to 17.9%, *p* = 0.040), without a significant change in the control group (from 20.5% to 17.6%, *p* = 0.12). No significant difference was found between the beginning (*p* = 0.59) and the end of the year (*p* = 0.036) in health behaviors of the participants.				Nutrition education Healthy food education Family education
**Bhave et al. [42]**	**School-Based Intervention** PA, diet, and general health, Increased extracurricular and intracurricular PA sessions (Daily yoga-based breathing exercises; making PA a ‘scoring’ subject) **Intensity:** NA	**Anthropometry:** Height, weight and waist circumference was recorded according to standard protocols **Physical fitness:** 1 min of sit-ups, 1 min of push-ups, a measured vertical jump, a measured long jump, a stand-and-reach test, a timed 30-m sprint **Diet and activity:** self-completed questionnaires	**BMI (kg/m^2^):** Pre and post, Boys: Mean, 17.1–22.3 Girls: Mean, 17.7—22.8) Children were fitter than controls in running, long jump, sit-up and push-up tests (*p* < 0.05 for all). The intervention did not reduce BMI (kg/m^2^) or the prevalence of overweight/obesity, but waist circumference was lower than the control group (*p* = 0.004).				Nutrition education Healthier school meals Health and nutrition education for teachers, pupils, and families
**Brown et al. [43]**	**A school-based pilot intervention** 90-min, 10-week primary school intervention PA and HE education PA activity sessions **Intensity:** NA	**Weight:** SecaTM 899 digital scales **Height:** Seca Leicester Height Measure stadiometer **BMI** = kg/m^2^	**Effects of program on BMI-SDS from pre to post**		***p***		Behavior change and parental engagement
			Pre, mean (SD): 0.49 (1.16)		*p* < 0.001		
			Post, mean (SD): 0.47 (1.17)				
			BMI-SDS: −0.03 (0.29)				
			Standardised BMI (BMI-SDS) scores were significantly decreased in the whole (*p* < 0.001).				
**Carlson et al. [44]**	**Type: Classroom PA breaks** 10 min of PA break **Intensity (min/day):** MVPA	**PA:** Actigraph GT3X+ accelerometers **Students’ behaviors:** Teacher survey	**Physical activity breaks min/day:** Fall 2013, mean, 5.2; Spring 2014, mean, 6.4 Class activity breaks were positively associated with MVPA of children (βs = 0.07–0.14; *p* = 0.012–0.016). Class-based PA practices can increase students’ level of PA throughout the day. βs = Standardized regression coefficient score				Teacher education
**Eather et al. [45]**	**Fit-4-Fun Program** 8-week physical education lesson (60 min/week) 8-week home activity (3*20 min per week) 8-week daily break-time activity program (recess, lunch) **Intensity:** NA	**Cardio-respiratory fitness (CRF):** 20 m shuttle run **Other tests: BMI (kg/m^2^)**, Y stage sit-up test, push-up test, basketball throw test, standing jump, sit and reach and pedometer	**CRF:** Mean, 1.14 levels, *p* < 0.001 **BMI:** Mean, −0.96 kg/m^2^, *p* < 0.001 **BMI z-score (BMIz):** Mean, −0.47, *p* < 0.001 **Flexibility:** Mean, 1.52 cm, *p* = 0.0013 **Muscular fitness:** Mean, 0.62 stages, *p* = 0.003 **Physical activity:** Mean, 3253 steps/day, *p* < 0.001 A school-based intervention focusing on fitness education significantly improved health-related fitness and PA levels in children.				Family engagement Home program
**Grydeland et al. [46]**	**Multiple interventions** 10 min of PA in classrooms Sports equipment for recess activities Pedometer **Intensity:** NA	**Height:** Wall-mounted measurement tape **Weight: **Digital body composition analyzer **BMI = kg/m^2^** **Waist circumference (WC):** A measuring tape between the lower rib and the iliac-crest	Beneficial effects were found for BMI (*p* = 0.02) and BMIz (*p* = 0.003) in girls, but not in boys. There were no intervention effects for WC and weight status (*p* > 0.05).				Home/parents activities Lessons with student booklet Posters for classrooms Fruit and vegetable (FV) break
**Hollis et al. [47]**	**‘PA 4 Everyone’ intervention** **School curriculum:** Interventions that maximize the level of PA in lessons and school **School environment:** PA support during breaks and lunch breaks **Intensity:** NA	**Socio-demographic characteristics:** Survey **Weight:** A portable digital scale (Model no. UC-321PC, A&D Company Ltd., Tokyo, Japan). **Height:** A portable stadiometer (Model no. PE087, Mentone Educational Centre, Springvale, VIC, Australia). **BMI = kg/m^2^** **PA:** Accelerometer (Actigraph GT3X+ and GT3X models, Pensacola, FL, USA43).	**Difference in change between groups**		***p***		Environment and family involvement Partnership and services: Activities involving the region and the family
			BMI (Baseline to 12 month): −0.28		0.012		
			BMIz (Baseline to 12 month): −0.05		0.130		
			There were group-by-time effects for weight and BMI (kg/m^2^) (*p* < 0.01) in favor of the intervention group, but not for BMIz (*p* = 0.13). School-based intervention achieved moderate reductions in adiposity among adolescents.				
**Kipping et al. [48]**	**The Active for Life Year 5 (AFLY5) intervention** Indirect intervention ways **Intensity:** NA	**MVPA, sedentary behavior:** Accelerometer **Weight:** Digital scale **Height:** A portable Harpenden stadiometer **Waist Circumference:** Flexible tape **Daily fruit and vegetable consumptions:** A Day in the Life Questionnaire	**Mean differences**		***p***		Teacher training Provision of lesson Child-parent interactive homework plans Preparation of school environment
			The differences in mean: −1.35 min/day		0.050		
			BMIz Scores: −0.02		0.41		
			Waist circumference: −0.12		0.03		
			School-based intervention is not effective at increasing levels of PA, but it is effective at BMI and waist circumference.				
**Kriemler et al. [49]**	**A multi-component PA program** 2 × 45 min/week: physical education lessons given by physical education teacher 3 × 45 min/week: physical education lessons given by classroom teacher Several 5-min short PA breaks 10 min/day: PA homework **Intensity:** NA	**Primary outcomes:** **Skinfolds (mm):** Harpenden calipers (HSK BI, British Indicators). **Aerobic fitness:** 20 m shuttle run **PA:** Accelerometer (MTI/CSA 7164, Actigraph, Shalimar, FL, USA). **Quality of life:** health questionnaire Body mass index **A cardiovascular risk score:** An automated oscillograph	**Intervention and control group comparison**		***p***		
			The z score of the sum of four skinfolds: −0.12		0.009		
			Z scores for aerobic fitness: 0.17		0.04		
			MDVA physical activity: 0.92		0.003		
			A school-based PA intervention improved PA and fitness and reduced adiposity in children.				
**Li et al. [50]**	**A multi-component PA intervention** PE improvement Extracurricular PA for students **Intensity:** 30 min MVPA (64–94% of their age-predicted max. heart rate).	**Weight:** A lever scale **Height:** A stadiometer **BMI = kg/m^2^** **Duration of MVPA:** Self-administered questionnaires	The reduction of BMI was statistically significant in the intervention group (−0.02 ± 0.06 kg/m^2^), compared with the increase of BMI in the control group (0.41 ± 0.08 kg/m2) (p < 0.001). The change in duration of MVPA in the intervention group (8.9 ± 4.3 min/day) was significantly different from that in the control group (−13.8 ± 3.3 min/day).				PA at home
**Lynch et al. [51]**	**Let’s Go! 5-2-1-0 Curriculum** (5) Fruits and Vegetables (2) Hours or Less of Recreational Screen Time (1) Hour of PA (0) Sugary Drinks 9 Hours of Sleep & Healthy Breakfast 7 Portion Sizes & Healthy Snacks 8 Wrap Up/Review **Intensity:** NA	**Healthy Habits:** Survey **BMI = kg/m^2^, median (Q1, Q3)** **PA:** Pedometer (The Omron HJ-321 pedometer).	**Intervention Group**	**Control Group**		***p***	Curriculum
			BMI (kg/m2, median change: 0.2	BMI (kg/m2, median change: 0.1		0.469	
			Number of pedometer steps per day (median): 2293.5	Number of pedometer steps per day (median): 2651.3		0.929	
			There was no statistical difference in the improvement of healthy habits, BMI, or PA in the intervention group compared with the control group (*p* > 0.05).				
**Madsen et al. [52]**	**Energy Balance for Kids with Play (EB4K with Play)** Intervention schools received one part-time RD coach and one full-time Playworks coach for 2 school years **Intensity:** NA	**PA:** Actigraph GT1M or GT3X accelerometer **Cardiorespiratory fitness:** 1-mile run **Anthropometric measures:** Standardised height and weight measurement **Other variables:** Fruit and vegetable consumption, dietary behaviors, dietary knowledge	**Intervention and control group comparison adjusted difference**		***p***		Collaboration with school personnel and families
			School-day PA: −0.1		*p* > 0.05		
			BMIz scores total: −0.07		*p* > 0.05		
			There were no group differences in change in PA or dietary behaviors, although BMIz decreased overall by −0.07 (*p* = 0.05).				
**Magnusson et al. [53]**	**School-Based PA Program** Organized field trips Promotion of active commute to and from school One extra class of physical education **Intensity:** NA	**Body fat percentage:** A dual-energy x-ray scan (DEXA). **Cardiorespiratory fitness (W/kg):** A Monark ergometer bike	**Intervention and control group comparison z scores**		***p***		Teacher training Outdoor teaching
			Cardiorespiratory fitness (W/kg: 0.37		*p* = 0.18		
			Waist circumference (cm): 0.15		*p* = 0.53		
			Skinfolds (mm): 0.10		*p* = 0.52		
			Body composition was not statistically significant (*p* > 0.05). Children in the intervention group increased their fitness by an average of 0.37 z score units more than the controls (*p* = 0.18). There was no significant difference in waist circumference and skinfold results (*p* > 0.05).				
**Shore et al. [54]**	**Step-Count Promotion ** Self-monitoring through pedometer use Self-monitoring Goal-setting strategies Benefits of an active lifestyle Overcoming barriers to achieve an active lifestyle Social support PA replacing sedentary behavior Components of fitness. **Intensity:** NA	**PA:** SW-401 DIGI-walker pedometer. **Fitness Performance:** Curl ups, shuttle run, endurance 1-mile run/walk, pull-ups, and sit and reach	**Step-count (steps/d) Intervention Group**	**Step-count (steps/d) Control Group**			Curriculum
			Pre-test Mean (SD): 9692 (476) Post-test Mean (SD): −12,307 (679)	Pre-test Mean (SD): 9420 (446) Post-test Mean (SD): −10,608 (702)			
			Step-count promotion program significantly increased daily accrued step counts versus control (*p* < 0.05). Shuttle and mile run performance decreased from pre- to post-intervention in both groups.				
**Sigmund et al. [55]**	**PA Intervention program** 20-min recess with PA content (in gym/school playground) PA (playing) undertaken during after-school nursery (40 min to ≤ 90 min) 2–3 short breaks per day **Intensity:** NA	**PA:** Caltrac accelerometer and Yamax Digiwalker SW-200 pedometer	There was a significant increase in daily activity levels compared to the control group (from 1718 to 3247 steps per day; and from 2.1 to 3.6 Kcal/Kg per day). The school-based intervention allows children to take more than 10,500 steps and reduces the risk of declined PA.				
**Thivel et al. [56]**	**PA program:** 120 min (two times for 60 min) of supervised physical exercise in addition to 2 h of Physical Education classes per week. **Intensity:** NA	**BMI (kg/m^2^):** A portable digital scale **Height:** A standing stadiometer (Seca model 720, Germany). **Waist circumference:** A level midway between the last rib and superior iliac crest. **Cardiorespiratory fitness:** The 20-m shuttle run test **Peak power:** A calibrated friction loaded ergometer.	**PA levels at pre and post-test (Intervention)**	**PA levels at pre and post-test (Control)**			
			Aerobic (stage) pre-test mean (SD): 3.10 (0.9) Aerobic (stage) pre-test mean (SD): 3.68 (1.1)	Aerobic (stage) pre-test mean (SD): 3.15 (1.14) Aerobic (stage) pre-test mean (SD): 3.31 (1.4)			
			Anaerobic and aerobic fitness levels were significantly improved in both lean and obese children (*p* < 0.05), but there was no significant change in anthropometric variables (*p* > 0.05).				
**Tian et al. [57]**	**PE intervention program** 7 entails various PF activities (weeks 1–6) and indigenous games (weeks 7–12). **Intensity:** 30 min MVPA aerobic exercise	**PA Levels:** The Children’s Leisure Activities Study Survey (CLASS) questionnaire **Bodyweight:** A portable electronic scale **Height:** Stadiometer Percentage of body fat was calculated from triceps and subscapular skinfolds measurements	**PA levels at pre and post-test (Intervention)**	**PA levels at pre and post-test (Control)**			Curriculum
			Total PA (min/week) pretest mean (SD): 91.0 (43.9) Total PA (min/week) post-test mean (SD): 112.1 (66.4)	Total PA (min/week) pretest mean (SD): 93.7 (52.2) Total PA (min/week) post-test mean (SD): 92.4 (55.4)			
			No significant difference between intervention and control groups at pre and post-test measurements (*p* > 0.05). Moderate PA, vigorous PA and total PA significantly increased in the experimental group after the 12-week intervention program.				
**Vander Ploeg et al. [58]**	**APPLE School** To improve healthy living habits of students **Intensity:** NA	**PA Levels:** The Omron HJ-720 ITC time-stamped pedometer	**Step-count (steps/d) APPLE Schools (2009)**	**Step-count (steps/d) APPLE Schools (2011)**			Versatile program
			School days, mean (SD): 11,371 (3306) School hours, mean (SD): 850 (233)	School days, mean (SD): 13,375 (3653) School hours, mean (SD): 933 (222)			
			Children were significantly more active in 2011 in comparison with 2009 (*p* < 0.001). Comprehensive school programs affect the levels of children’s PA during school.				

## Supplementary Materials

Supplementary materials can be found at https://www.mdpi.com/1660-4601/17/1/347/s1, Table S1: Quality assessment of 19 studies, File S2: Examples of Search Strategy.
